# On Medical Provision for Rail Roads, as a Humanitarian Measure, as Well as a Source of Economy to the Companies

**Published:** 1863-01

**Authors:** 


### EDITORIAL DEPARTMENT.

*On Medical Provision for Rail Roads, as a Humanitarian Measure, as well as a source of Economy to the Companies. In two papers, read respectively before the New York State Medical Society, February 5th,* 1862, *and the Surgical Section of the New York Academy of Medicine, October 28th,* 1862. *By* EDMUND S. F. ARNOLD, M. D., *Jefferson College, Philadelphia, Member of the Royal College of Surgeons of England, Resident Fellow of the New York Academy of Medicine, of Yonkers, N. Y. New York:* BAILLIERE BROTHERS, 440, *Broadway.*

On the 28th of February last, Dr. Arnold was called upon to explain his views, of which the following may be regarded as a summary: "The attachment to our leading stations, at intervals, where practicable, of not more than ten miles, of a small room kept at all times in readiness for the reception of injured persons, and providing the same with surgical apparatus, carrying also appliances in the cars where the stations were too far apart to enable us to get at such apparatus without great loss of time.—The appointment of competent surgeons to attend to the injured, unpaid, but payable for actual services rendered; such services to extend, however, only to such a period as shall enable sufferers to be removed without risk to the care of their own medical attendant, or be otherwise safely disposed of. The district surgeon would also take authoritative charge in case of any great accident involving injury to a number, seeing to it that all were efficiently cared for, to effect which he would be empowered not only to avail himself of any efficient medical assistance at hand, but also to call in additional aid, if necessary. The appointment, lastly, of a general medical superintendent to supervise the working of the whole."

By the adoption of such arrangements, with their attendant details, he claims, not only that the loss of much life and limb would be avoided, but that, in cases where thus much could not be accomplished. we should at least be enabled greatly to mitigate the sufferings of the victims of railway casualty."

"A Bill to provide Compensation to Passengers for personal injury received on the Railroads of the State, and for the establishment of Surgical Stations and Hospital Accommodations on the Railroads of the State,"

was introduced by Senator Smith, of Kings County, on the 5th of February last, and on the following day referred to the Insurance Committee. Its main provisions in its present state are as follows:

It provides for an association of the railroad companies of the State, the same to be a "body corporate," managed by a "Board of Managers," consisting of the Presidents, or such other officers of the associated companies, as may be designated by the respective companies, and the President of the association, who shall be a citizen of the State of New York, and not an officer of any railroad company.

The association shall make up a guarantee fund of $100,000, chargeable upon each road as to its passenger traffic; and to enable the association of railroads to meet casualties, the respective companies shall, in their discretion, be allowed to charge four-tenths of a mill per mile to every passenger, or one cent for every twenty-five miles or distance within it, in addition to the usual fare. In return for this, each passenger is guaranteed, in case of death, $5,000 to his heirs; in case of loss of a limb, or an incurable injury seriously interfering with usual occupations, $5,000, and for minor injuries $25 per week, provided that such payments shall not extend over fifty-two weeks. The association also undertakes to establish surgical stations, at distances of not over ten miles from any one spot, which shall be provided with suitable necessaries, and to appoint competent surgeons to attend them when required. This done, the railroad companies are to be exempted from all liability on account of any accident to passengers.

The bill provides further, that the fund raised by the tax upon passengers, which may be called the "Casualty Fund," shall be employed for no other purpose than to pay compensations with the necessary expenses of management, and that whatever remains over and above shall accumulate, and when the interest on such accumulation shall amount to a sum equal to tne tax of one-tenth of a mill, then the tax on the public shall be reduced to three-tenths of a mill, and so on until the passenger tax is abolished altogether. The medical provision will be paid for by other means, to be presently mentioned.

It provides that on an accident occurring on any road, the company shall be find to the extent of one-third of the amount to which it has rendered the associated fund liable. This fine is to go into a special fund which may be called the "Reward and Penalty Fund." For instance, if five passengers were killed and others injured so as between them to draw for five thousand more on the casualty fund, and the total thereby made $30,000, the company, on whose line the accident occurred, would pay $10,000 into the reward and penalty fund. Thus, while whatever comes from the public will go back to the public, on the other hand, the companies are by no means relieved from liability. Owing to the liberal compensations allowed, the penalty would in many cases be greater than that now entailed through the instrumentality of courts of law.

A clause has likewise been introduced by which, when companies send sufferers to hospitals, and pay usual rates for attendance on the same, such action shall not be held to imply any legal liability for damages on the part of the company. The object of this is as follows: when a person is injured and sent by the railroad company to an unendowed hospital, it (the company) is willing to pay the same as others for the attendance, but insists that a paper shall be signed by the injured person to the effect that doing so shall not be held as an admission of culpability on its own part. No sooner are such persons in the hospital than they are surrounded by a low class of lawyers, who persuade them not to sign any such paper, and consequently companies are compelled to refuse payment for their own protection. The difficulty is obviated by the above clause.

Out of the reward and penalty fund all charges of medical provision and general hospital expenses are to be paid, and at the end of the fiscal year whatever remains over and above is to be re-distributed among all the companies pro rata as to their contribution to the casualty fund. Thus, as Mr. Morgan observes, "rewards and penalties are set forth of the highest importance, as securing care and proper equipment on every road of the association." Companies not meeting with any accidents will be absolute gainers, while those with whom they occur will foot all the expenses."

Such are the main provisions of the Bill.

The attention of the State Legislature has been called to this subject, and it seems thus far to have met with general favor both by the medical profession and by all interested. The object mainly seems to be, to provide proper surgical attendance in case of railroad accident, and protect the unfortunate victims of such disaster from the imposition of quacks who often times intrude their services, when total neglect would be comparatively an infinite blessing; and to make suitable provision for payment of expenses and damages, without long, tedious and expensive litigation. It is something upon the principle of our marine regulations, requiring every sailor to pay hospital fees; so this bill proposes that every passenger pay a small per cent., and if injured be immediately and properly attended, and damages cheerfully and promptly paid.

So far as all this goes it is a commendable and praiseworthy object, and every well wisher of the race would favor its adoption. There is only one suggestion which we desire to make in the premises, as the details have been very fully and systematically considered. "Railroad corporations," or "Boards of Managers," are about as liable to employ or appoint incompetent men to places of highest trust and responsibility, upon recommendation of interested parties, as political corporations, and if our railroad accidents are to be directed by men selected as all our political offices are filled, then nothing would be gained, and something might be lost. An energetic and capable man might by accident be called to direct and care for persons thus injured, while if the usual influences were allowed, which control all our political appointments, incompetency and stupidity would constitute the rule, and poor humanity might be well and promptly paid, yet worse treated *after* the accident than *by* it.

We hope to see this subject fairly considered and the details of the plan matured, so as to make it a great blessing, such as it is designed; and we have no hesitation in saying that if the plan proposed by Dr. Arnold is faithfully adopted and properly carried out, it will prove one of the greatest safeguards to the traveler which can possibly be instituted.

